# Patient Posture-Adaptive Non-Contact Respiratory Rate Measurement Inside a Moving Ambulance Using RGB and Thermal Cameras

**DOI:** 10.3390/diagnostics16193244

**Published:** 2026-10-08

**Authors:** Daiga Nemoto, Nozomi Takahashi, Fumitaka Mukouyama, Taka-aki Nakada, Yukihiro Nomura, Toshiya Nakaguchi

**Affiliations:** 1 Department of Medical Engineering, Graduate School of Science and Engineering, Chiba University, 1-33, Yayoicho, Inage-ku, Chiba-shi 263-8522, Chiba, Japan; 2 Department of Emergency and Critical Care Medicine, Graduate School of Medicine, Chiba University, 1-8-1, Inohana, Chuo-ku, Chiba-shi 260-8670, Chiba, Japan; 3 Department of Obstetrics and Gynecology, Chiba University Hospital, 1-8-1, Inohana, Chuo-ku, Chiba-shi 260-8677, Chiba, Japan; 4 Center for Frontier Medical Engineering, Chiba University, 1-33, Yayoicho, Inage-ku, Chiba-shi 263-8522, Chiba, Japan

**Keywords:** non-contact respiratory rate measurement, RGB camera, thermal camera, image registration, nostril detection, ambulance monitoring

## Abstract

**Background/Objectives**: Monitoring and evaluating respiratory rate (RR) plays a critical role during emergency transport. Conventional RR measurement methods have primarily relied on electrocardiogram-based approaches, which are susceptible to large errors caused by patient body movement and impose a burden on patients due to their contact-based nature. This study aimed to develop a non-contact RR measurement method using RGB and thermal cameras that can be applied inside a moving ambulance. **Methods**: One of the key challenges in non-contact RR measurement inside an ambulance is accommodating changes in patient posture. We addressed this by patient posture-adaptive automatic image registration between RGB and thermal cameras and developing a nostril detection method robust to posture changes. **Results**: The image registration error of the proposed method was evaluated at various distances from the camera, and the error was confirmed to remain within 1 pixel. RR measurement experiments simulating various respiratory conditions were then conducted inside a moving ambulance. The average of mean absolute error (MAE) was 0.95, 0.59, and 2.80 breaths per minute (breaths/min) for bradypnea, normal, and tachypnea, respectively, in the supine position, and 1.08, 1.90, and 4.38 breaths/min, respectively, in the semi-sitting position. Evaluation of the proposed nostril detection method further confirmed improved RR estimation accuracy compared with the conventional method. **Conclusions**: This study achieved patient posture-adaptive automatic image registration and robust nostril detection and successfully conducted experiments under realistic and challenging conditions, representing a substantial step toward the real-world implementation of a non-contact RR measurement system in ambulances.

## 1. Introduction

Emergency transport patient assessment requires the quick identification of signs of worsening conditions within limited time and environmental constraints, enabling prompt transport to the appropriate medical facility. This is critical for patients with mild-to-moderate conditions, who do not initially present with signs severe enough to warrant closer monitoring but may deteriorate suddenly and unpredictably during transport; for such patients, continuous vital-sign monitoring is especially valuable, since deterioration is difficult to anticipate from external appearance alone making objective, continuous monitoring essential [1]. Vital signs in patients during emergency transport are a very useful indicator for assessing their severity and outcome [2,3]. In clinical practice, respiratory rate (RR) is recognized as a critical vital sign [4,5]. However, it remains one of the least accurately measured vital signs. During ambulance transport, assessment relies on brief visual counting, a qualitative and subjective method that is inherently prone to measurement errors. Furthermore, the ambulance environment is characterized by continuous patient movement, vehicle vibration, and frequent changes in posture associated with ongoing medical procedures. Under such conditions, conventional RR measurement methods based on electrocardiographic signals are susceptible to motion artifacts and electrode displacement, which compromises the stability and accuracy of continuous RR measurement [6,7]. Moreover, the use of contact sensors inside ambulances is susceptible to interference from posture changes during transport, transfers to stretchers, and other procedures. This can impose additional burdens on patients experiencing pain, discomfort, or anxiety. Such environment-specific constraints prevent reliable measurement of RR. Consequently, this heightens the risk of failing to detect early signs of deterioration, such as abnormal RR, potentially delaying initial response, affecting destination selection, and influencing treatment decisions. Therefore, establishing a method capable of stable, non-contact RR measurement even within the unique environment of an ambulance is strongly required.

Various contactless techniques for RR monitoring have been proposed to overcome the limitations of conventional contact-based sensors. As comprehensively reviewed by Massaroni et al. [8], these methods include video-based techniques, thermal imaging, radar-based systems, and acoustic approaches, all aiming to enable unobtrusive RR measurement. Among these approaches, thermal imaging detects the temperature modulation at the nostrils or mouth that accompanies the respiratory cycle: inhaled air is close to ambient temperature, whereas exhaled air approaches body temperature. This provides a contact-free signal source that, unlike video-based approaches, does not depend on ambient illumination [9,10,11]. Owing to these characteristics, thermal cameras have been investigated across a wide range of settings, from clinical use, such as monitoring patients in intensive care units or neonatal units, to home care settings for managing chronic conditions such as chronic obstructive pulmonary disease [12].

Despite their potential, maintaining robustness to motion remains a major challenge for non-contact methods when applied individually, particularly in highly dynamic environments such as emergency transport. These observations suggest that single-modality non-contact approaches may be insufficient to ensure robust RR measurement under the dynamic conditions. Accordingly, multimodal sensing strategies that integrate complementary modalities warrant further investigation. We considered the integration of RGB imaging for anatomical tracking with thermal imaging for RR measurement to be a promising approach.

Several studies have proposed RR measurement methods using both RGB and thermal cameras. Hu et al. [13] proposed a method that combines landmark detection on RGB images with temperature signal extraction from thermal images and evaluated it using seated subjects in a tripod-fixed indoor environment. Maurya et al. [14] fixed the camera approximately 1.8 m away from the subjects and conducted the evaluation with seated subjects. Yang et al. [15] evaluated stationary subjects at 60–120 cm from the camera, while Khan et al. [16] evaluated seated subjects at 72–78 cm from the camera. As described above, existing studies have been limited to evaluations conducted in laboratory settings, where subjects sit or remain stationary while facing the camera. In an ambulance, however, patient’s posture varies from subject to subject, as it is adjusted according to the patient’s requests and condition. Furthermore, due to safety constraints, cameras must be positioned so as not to interfere with emergency medical activities inside the ambulance, which inevitably results in a certain distance from the subject, and ambulances are constantly subject to motion caused by driving. Existing studies have not fully addressed how to handle these conditions, nor have they been validated in the specialized environment of a moving ambulance.

This study aims to establish an RR measurement method using two cameras that can adapt to a moving ambulance, particularly accommodating changes in patient posture. Its effectiveness was evaluated through a robustness evaluation experiment in a moving ambulance. The novelty of the proposed system lies in the following three points: (1) an image registration method that eliminates the need for individual camera alignment for each patient and posture; (2) a method that performs stable ROI detection using a single algorithm across a variety of body positions, including supine and semi-sitting positions; and (3) evaluation of the RR estimation through measurement experiments conducted inside a moving ambulance.

## 2. Materials and Methods

**Figure 1:**
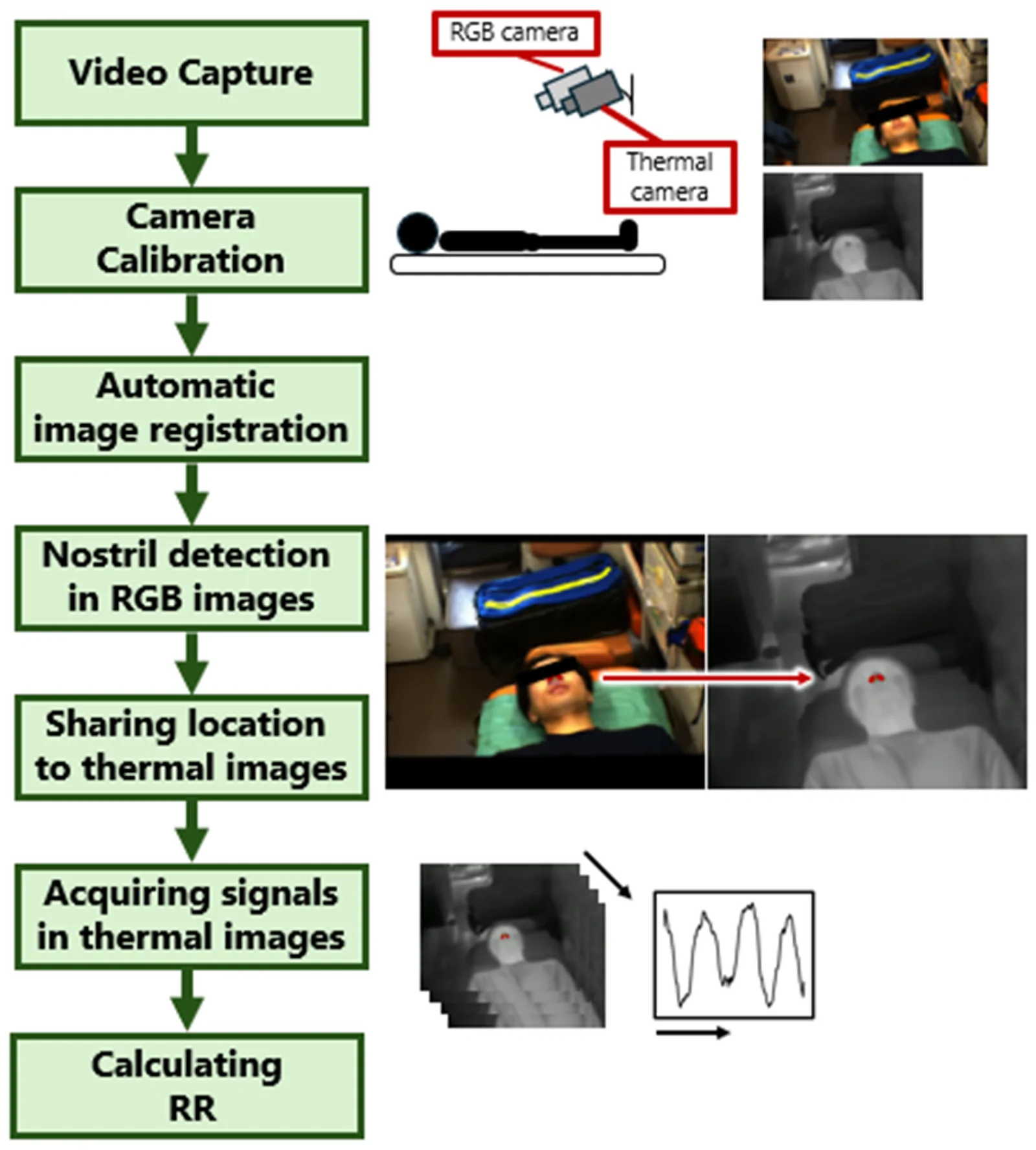
Overview of the proposed system.

In this study, we propose non-contact RR monitoring system using an RGB camera and a thermal camera in ambulance environments. The flow of the proposed system is shown in Figure 1. The following sections provide a detailed description of each component of the system.

### 2.1. Proposed Methods

This section consists of four components: camera calibration (Section 2.1.1), patient posture-adaptive automatic image registration (Section 2.1.2), nostril detection using the RGB camera (Section 2.1.3), and acquisition of the respiratory waveform from the thermal camera (Section 2.1.4). Among these, camera’s intrinsic parameter acquisition using a calibration board (Section 2.1.1) and the preparation stage of image registration (Section 2.1.2)—specifically, the acquisition of calibration board images—are performed outside the ambulance prior to deployment. All remaining processes are performed inside the ambulance, in accordance with the workflow shown in Figure 1.

#### 2.1.1. Camera Calibration

**Figure 2:**
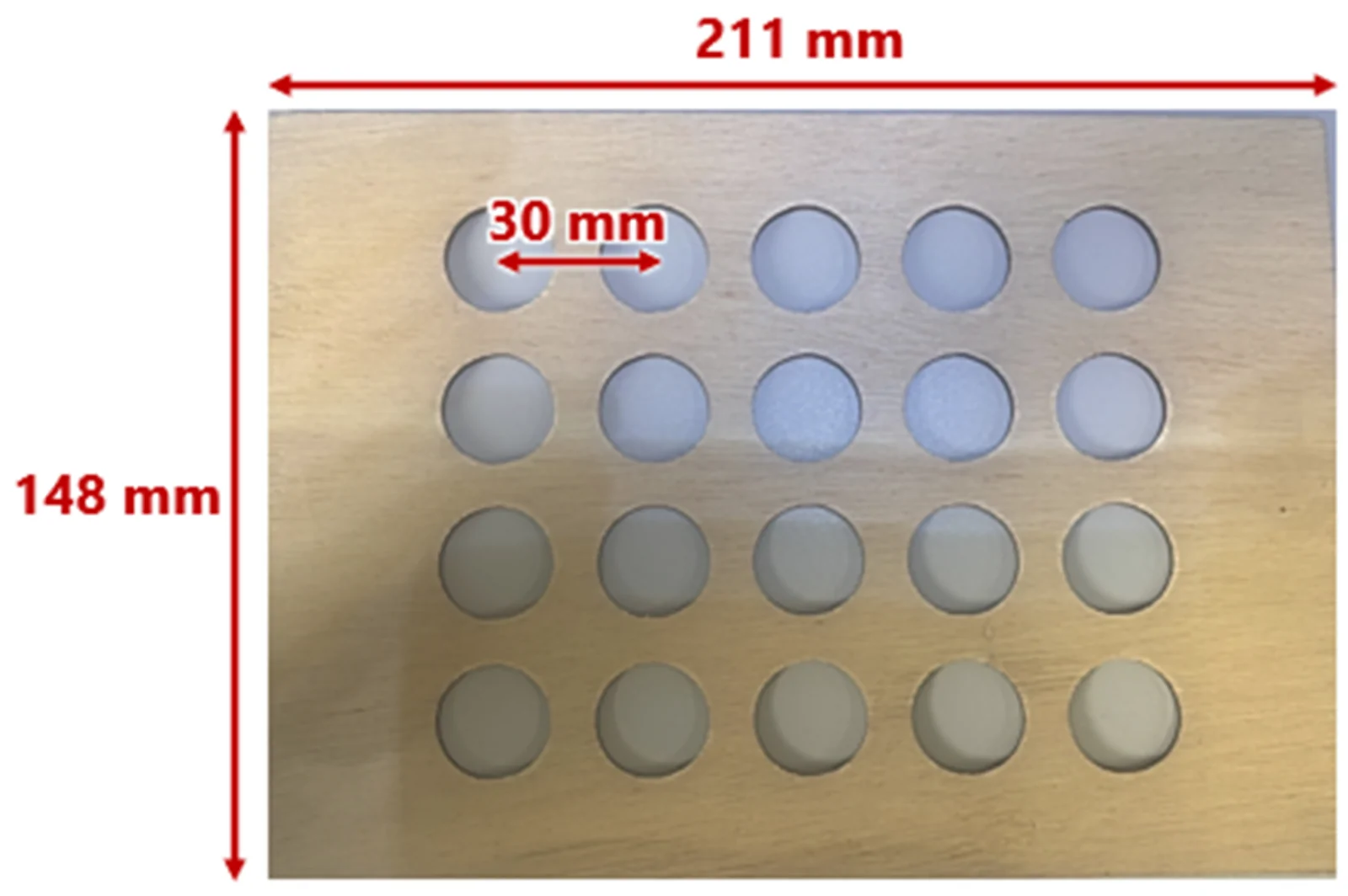
Calibration board. A grid of 20 circular cutouts, arranged in 4 rows and 5 columns with a center-to-center spacing of 30 mm, was drilled into a wooden board (148 mm × 211 mm × 2.5 mm).

Camera calibration is the process of determining a camera’s intrinsic parameters and correcting image distortion. This is typically performed using a chessboard pattern [17]; however, thermal cameras cannot detect chessboard corner coordinates due to the absence of thermal contrast between the black and white squares. To address this limitation, various heated calibration targets have been proposed, including circular patterns arranged on wooden or plastic boards [18]. In this study, a calibration board featuring a grid of circular cutouts was developed (Figure 2). The board was heated using a hair dryer, applied uniformly across its surface immediately before image acquisition. Heating the board creates a temperature difference against the background, making the circular grid points thermally visible. Images were captured promptly after heating, before the thermal contrast decayed. Grid point positions were then detected in both the RGB and thermal images using the circular grid detection function of OpenCV, in which each grid point was located as the centroid of the corresponding detected circular region. The intrinsic parameters and distortion coefficients of each camera were subsequently estimated from these correspondences using the method of Zhang [17].

#### 2.1.2. Patient Posture-Adaptive Automatic Image Registration

Respiration causes temperature changes around the nostrils due to the alternating flow of warm exhaled air and cooler inhaled air. Since the ambient air inside the ambulance is appropriately temperature-controlled, the inhaled air is assumed to remain below body temperature, making this region a key source of thermal signal for RR measurement. Because these temperature changes are subtle, precise localization of this region is necessary to reliably capture them as a signal. To this end, nostril detection is performed on the RGB image, as it provides higher spatial resolution and clearer facial features than the thermal image. However, since the optical axes of the RGB and thermal cameras are not aligned, the detected nostril position cannot be directly applied to the thermal image. In principle, optical axis alignment could be achieved using an optical setup such as a beam splitter, or half mirror. However, this approach is not adopted in this study due to constraints on device size, as well as the substantial risk that vibration during ambulance transport would progressively cause misalignment of the mirror angle. Therefore, image registration is required to align the coordinate systems of the two cameras, enabling the nostril position detected in the RGB image to be accurately transferred to the corresponding location in the thermal image.

As one such approach, image registration in this study is performed using a calibration board to obtain corresponding points between the RGB and thermal images [14,16], from which the homography matrix is estimated. This approach enables high-precision registration using stable reference points. However, conventional method using a calibration board assumes a fixed distance between the patient and the camera and does not account for situations where this distance changes. Under a perspective projection model, a change in this distance alters not only the image-space magnification but also causes a nonlinear shift in the radial pixel positions away from the optical axis, distorting the geometric configuration of feature points on the 2D image plane in a non-similar manner. Consequently, this method requires re-registration using the calibration board whenever the distance changes.

In an ambulance environment where the patient’s posture continuously changes, manual re-registration at every postural change is impractical. The proposed patient posture-adaptive automatic image registration method for the two-camera system therefore offers practical utility. The proposed method consists of two stages: a “preparation stage,” which must be performed manually once, prior to installing the cameras inside the ambulance, and an “automatic processing stage,” which is executed each time the patient’s posture changes after the cameras have been installed.

**Figure 3:**
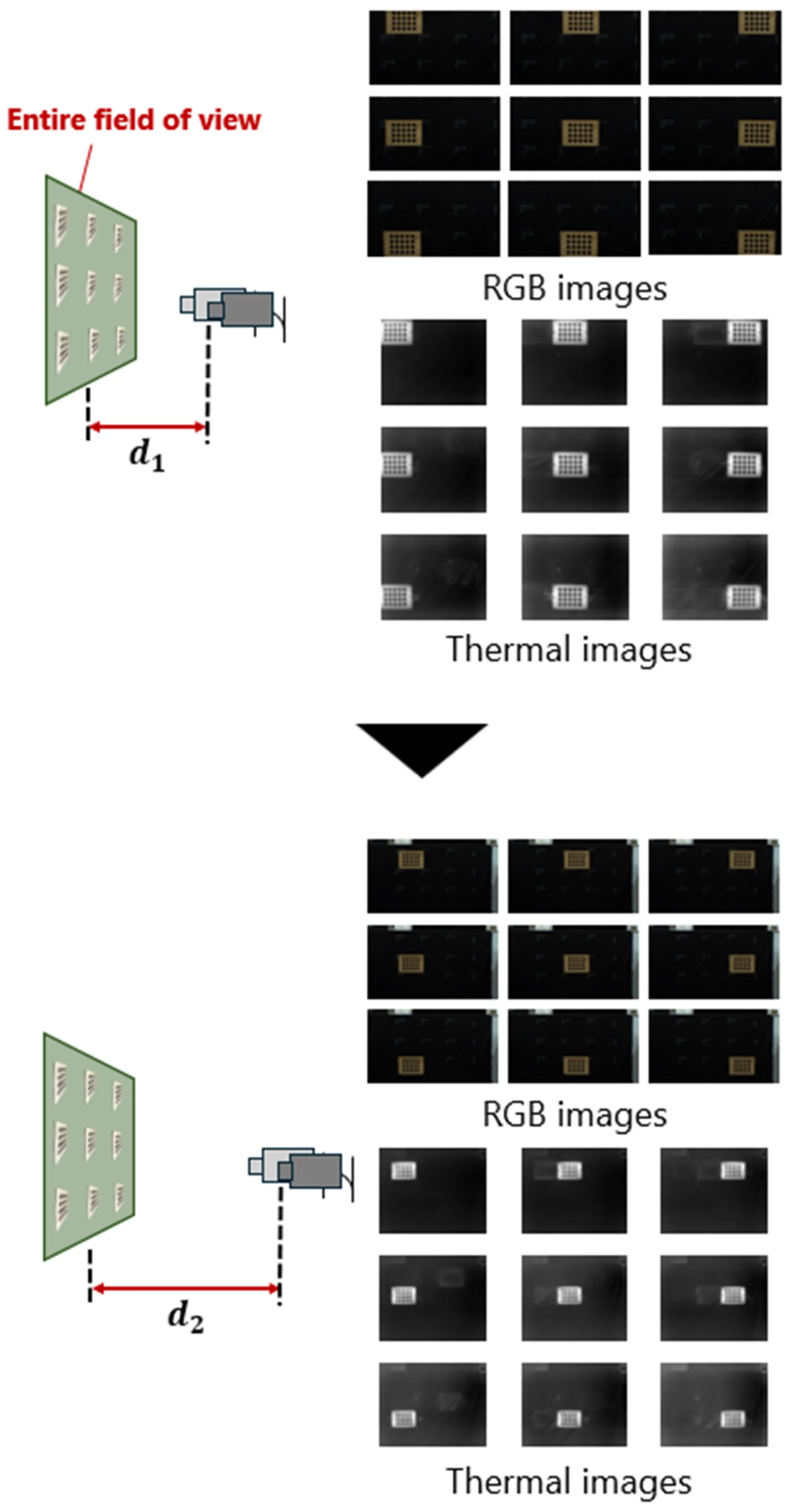
Preparation of the calibration board. The board is positioned to fill the entire field of view of the camera, and an image is first captured at the *d*_1_, followed by an image captured at the *d*_2_.

In the preparation stage, calibration board images must first be acquired using both the RGB and thermal cameras. First, the calibration board is placed at an arbitrary distance (*d*_1_), and images are captured with both cameras. At this distance, the board is positioned within a plane perpendicular to the optical axis of the camera. Since a single image captures only a portion of the field of view, images are acquired at multiple board positions within this plane so that the entire field of view is covered. Images are then captured at a second distance, *d*_2_, following the same procedure as described for *d*_1_. As a result, two corresponding image sets are obtained for each camera: one captured from *d*_1_ and one from *d*_2_ (Figure 3). This completes the preparation stage.

The automatic processing stage consists of the following three steps

1. Distance estimation between the patient and the camera.
2. Generation of a homography matrix corresponds to the estimated distance.
3. Image registration using the generated homography matrix.

For distance estimation, Depth Pro [19] was used. Depth Pro is a foundation model for zero-shot metric monocular depth estimation that predicts high-density, high-resolution depth maps with absolute scale from a single RGB image.

Next, grid points are detected in each of the previously prepared image pairs, separately for the RGB and thermal cameras. As the calibration board shown in Figure 3 was used in this study, each image contains a set of 20 grid points. Here, an image pair refers to two images—one captured at the *d*_1_ and one at the *d*_2_. The grid points position corresponding to the estimated distance are then generated through interpolation between the grid point positions detected in the *d*_1_ and *d*_2_ images of the same pair. This interpolation uses inverse-proportional interpolation, as formulated in Equation (1). This interpolation is applied to all 20 grid points within each image pair.

(1) $$p_{e,i}=p_{1,i}+\frac{d_{2}(d_{1}-d_{e})}{d_{e}(d_{1}-d_{2})}(p_{2,i}-p_{1,i}),$$

where *p*_1,*i*_ and *p*_2,*i*_ denote the (*x*, *y*) coordinates of the *i*-th grid point detected in the images captured at *d*_1_ and *d*_2_, respectively (*i* = 1, 2, …, 20); *d_e_* denotes the estimated distance between the patient and the camera; and *p_e_*_,*i*_ denotes the interpolated (*x*, *y*) coordinates of the *i*-th grid point at distance *d_e_*.

Finally, the homography matrix is generated using the grid points obtained through this interpolation. The resulting homography matrix is then applied to register the RGB image with the thermal image.

#### 2.1.3. Nostril Detection in RGB Camera

Nostril region detection is a necessary process for identifying the respiratory region of interest and transmitting its position information to the thermal camera. Conventional nostril detection methods typically defined facial landmark points surrounding the nostrils and used the detected landmarks to designate the respiratory region of interest. However, since only the nostril region exhibits temperature changes associated with respiration, these conventional methods suffer from reduced RR estimation accuracy when the defined region includes a substantial area other than the nostrils, which can occur depending on the subject’s posture. Conventional nostril detection methods could handle specific postures; however, they lacked robustness across arbitrary postural variations [13,14,15,16,20]. To address this limitation, a method for robust and pinpoint nostril detection was developed.

**Figure 4:**
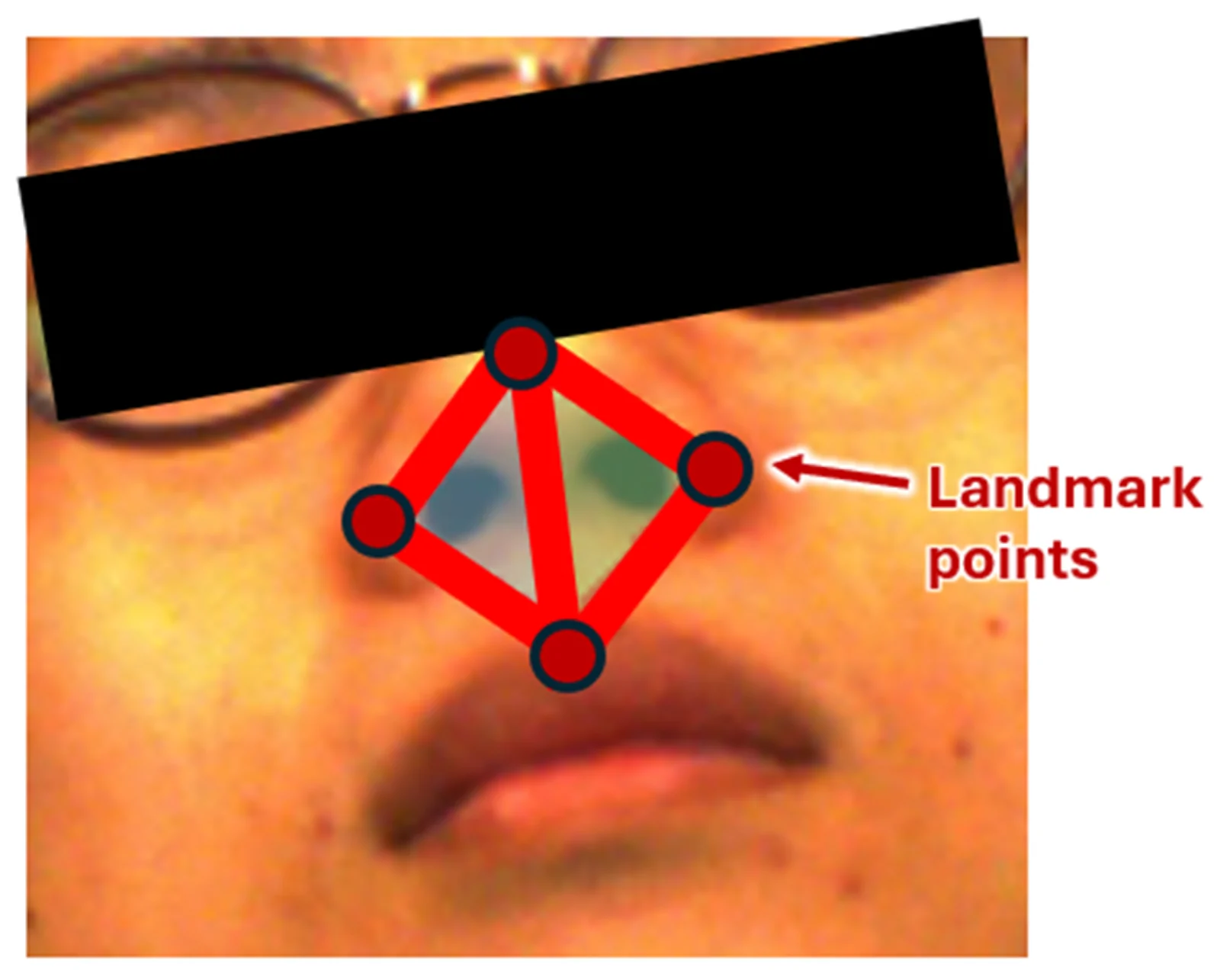
Creation of nostril-enclosing regions. Landmark points around the nostrils are detected using the RetinaFace model, and separate regions enclosing the left and right nostrils are created (the region enclosing the left nostril is shown in blue, and the region enclosing the right nostril is shown in green).

First, landmark points around the nostrils are detected. In this study, the RetinaFace model [21] was used for landmark detection, as it is robust to variations in head pose. The detected landmark points are used to define bounding regions containing the left and right nostrils, respectively (Figure 4). Any landmark points that enclose the left and right nostrils may be used for this purpose.

**Figure 5:**
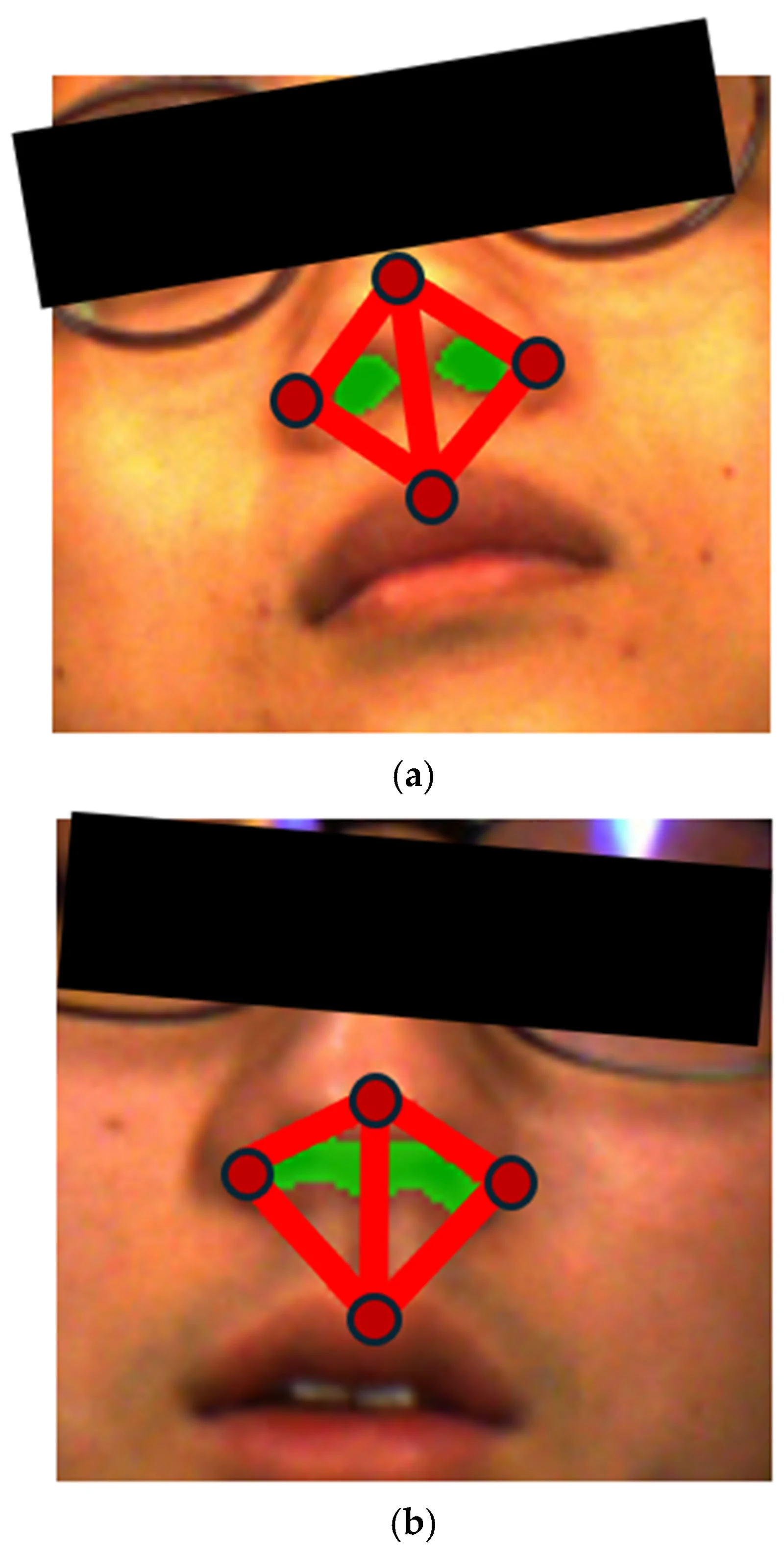
Examples of nostril detection from the left and right regions. The green areas indicate the detected nostril regions. (**a**) A subject in which the nostrils were pinpointed successfully. (**b**) A subject in which the nostrils were not pinpointed successfully; in both the left and right regions, an area including both the nose tip and the nostril was falsely detected as the nostril region.

Next, Otsu’s thresholding [22] is applied separately to the left and right regions, exploiting the difference in brightness between the nostrils and the surrounding area. At this stage, however, each of the initially defined regions may contain other areas with brightness similar to that of the nostrils, such as eyeglass frames, which can be falsely detected as nostril candidates after Otsu’s binarization. As a result, regions other than the nostrils may also be detected within each of the left and right regions. To address this issue, the product of circularity and area is computed for all candidate regions, and the region with the highest value is selected as the nostril region. Since the nostrils are approximately circular and have a certain minimum area, this metric allows them to be readily distinguished from falsely detected regions. This calculation is performed independently for the left and right regions, yielding one selected region for each side (Figure 5).

**Figure 6:**
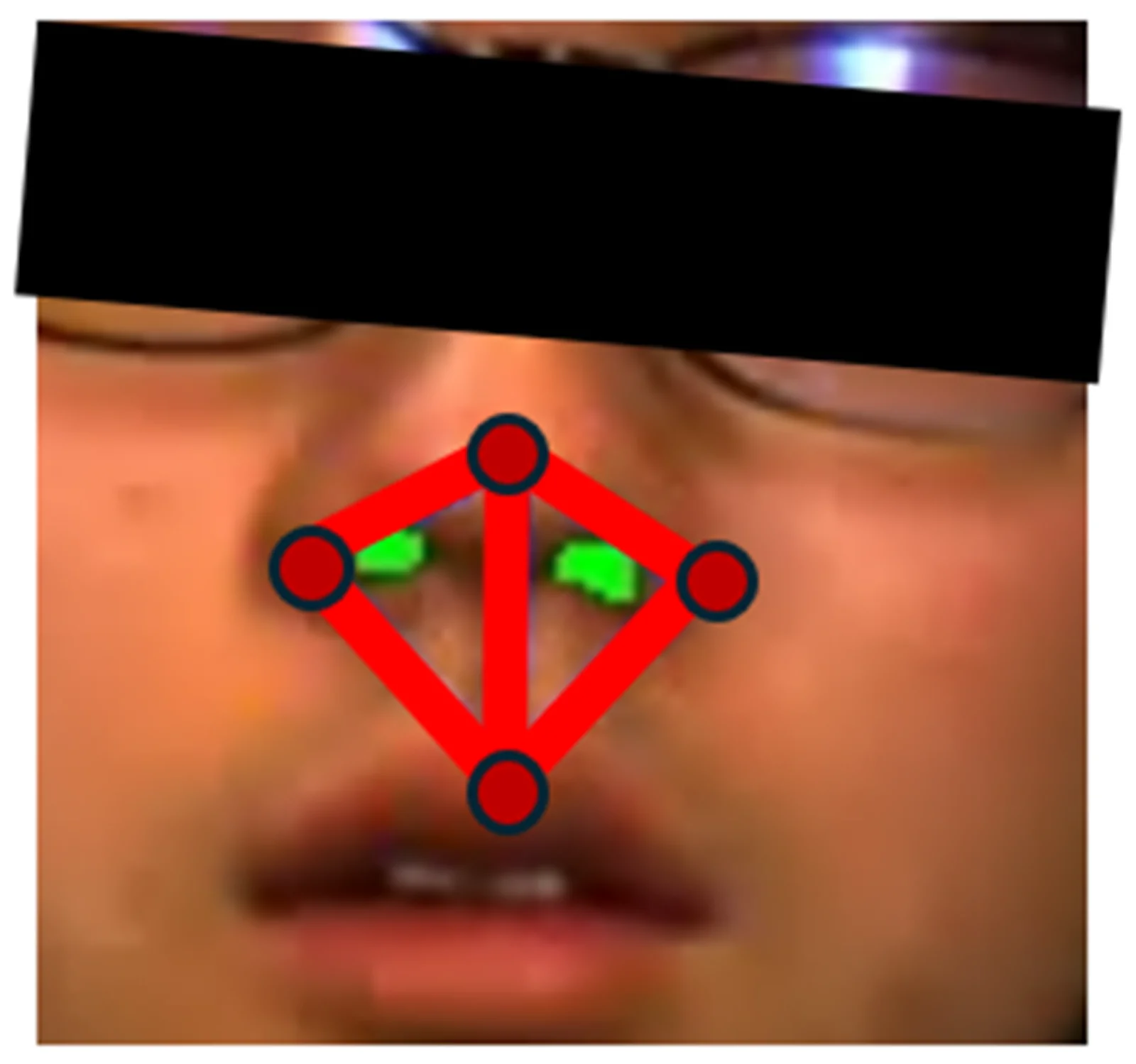
Final pinpoint nostril detection result for the subject shown in Figure 5b.

After determining one region for each side, the two regions are compared. If the two regions are not in contact with each other like Figure 5a, nostril detection is considered to have been successfully pinpointed. However, depending on the subject’s posture, the tip of the nose may cast a shadow with a brightness similar to that of the nostrils, causing the detected region to include the nose tip like Figure 5b. This can result in the two regions merging into one. To distinguish the nose tip from the nostrils, Otsu’s thresholding is applied again to the current region. The threshold is then progressively lowered until the region splits from one into two, ultimately achieving pinpoint detection of the nostril regions (Figure 6).

#### 2.1.4. Acquiring Respiratory Waveform from a Thermal Camera

The nostril position information obtained from the RGB image is shared with the thermal camera. Since the two camera images have already been image registered, it is sufficient to simply transfer the coordinates of the nostril regions detected in the RGB image to the thermal image. For each frame, the mean pixel value within the pinpointed nostril region is then calculated and used as the signal value for that frame. A respiratory waveform is subsequently constructed from the signal values obtained across all frames. To facilitate RR estimation, linear detrending and a third-order Butterworth low-pass filter with a cutoff frequency of 0.5 Hz are applied to the waveform; this cutoff frequency was selected to cover the typical range of RR in adults. Finally, zero-crossing points are identified, and the median interval between consecutive zero-crossing points is used as the representative value for calculating the RR, as formulated in Equation (2).

(2) $$RR=\frac{60}{2\cdot \Delta t_{\mathrm{med}}},$$

where Δ*t_med_* denotes the median of the intervals between all consecutive zero-crossing points, including both rising and falling zero-crossings, and *RR* denotes the estimated RR in breaths per minute.

### 2.2. Experimental Setup

The experiment was conducted inside a moving ambulance operated within the grounds of Chiba University Hospital, along a route including straight sections, turns, and stops, at speeds typical of low-speed driving on hospital premises. No emergency medical procedures were performed in the cabin during the measurements. Measurements were conducted during daytime transport, and the illumination on the subject’s face was not controlled; it varied naturally over the course of each measurement, owing to sunlight entering through the vehicle windows.

Eight healthy subjects were recruited for the study, comprising two females and six males, aged 21–23 years (mean ± SD: 21.9 ± 0.8 years). This study was approved by the Institutional Review Board of the Graduate School of Medicine, Chiba University (approval number: 2733, approved on 8 September 2022).

**Figure 7:**
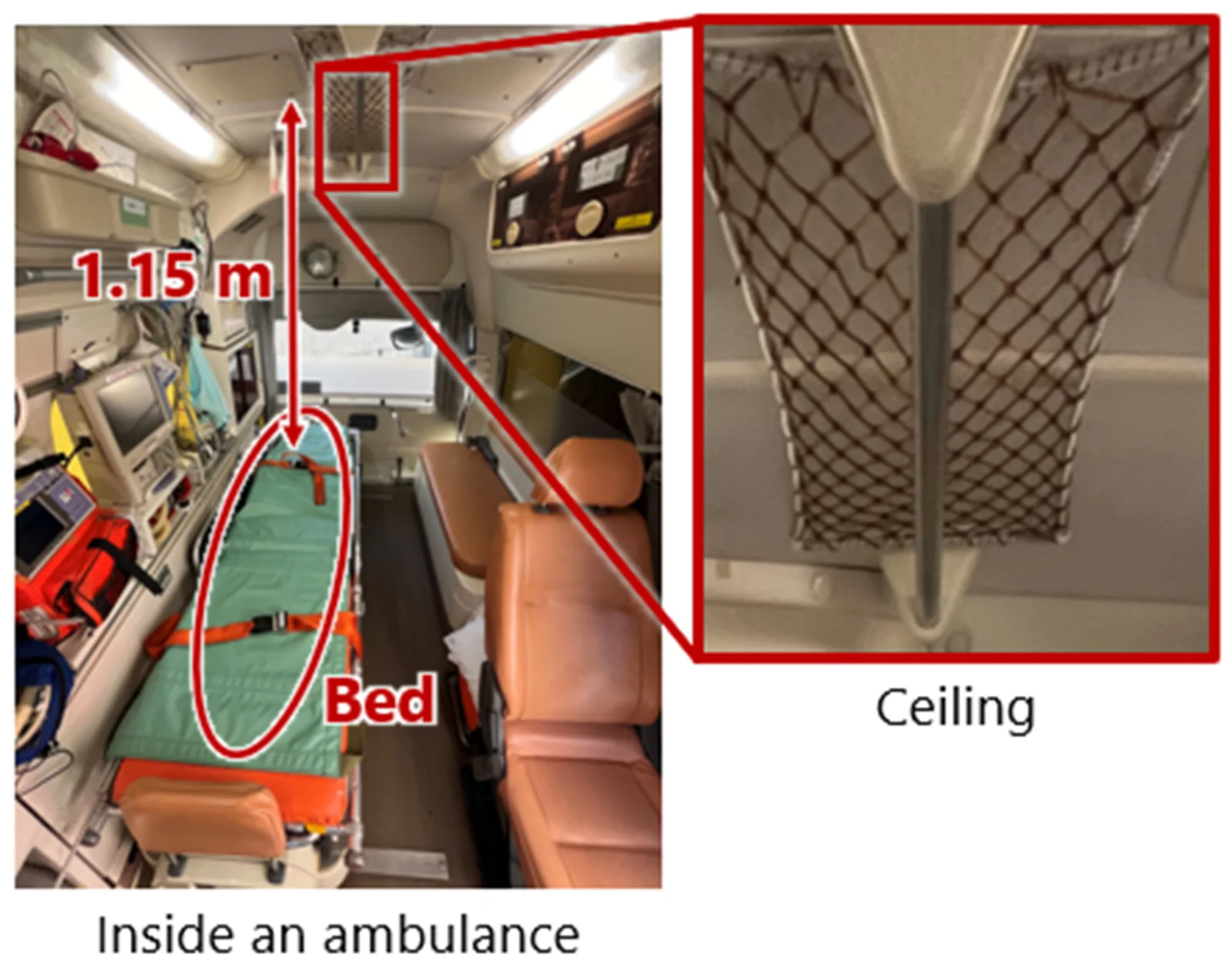
Experimental environment. The distance from the ceiling to the bed inside the ambulance is 1.15 m. A pole is mounted on the ceiling, to which the RGB and thermal cameras can be fixed.

Two cameras were used for data acquisition: an RGB camera (UI-3860CP-C-HQ Rev. 2, IDS Imaging Development Systems GmbH, Obersulm, Germany) with a resolution of 1936 × 1096 pixels, equipped with an 8 mm C-mount lens (M0814-MP2, Computar, CBC Co., Ltd., Tokyo, Japan) set to an aperture of F/8, and a thermal camera (20640A032-6PARX, Boson, Teledyne FLIR LLC, Goleta, CA, USA) with a resolution of 640 × 512 pixels, a 14 mm lens (32° horizontal field of view). The two cameras were operated independently and mounted on a custom 3D-printed fixture designed to maintain parallel optical axes with a fixed baseline distance of 34 mm between the two camera centers. The camera assembly was installed on the ceiling of the ambulance to avoid interfering with emergency medical activities inside the ambulance (Figure 7). Patients are loaded onto the stretcher head-first, ensuring that the head always remains oriented toward the front of the vehicle. Even in emergency situations, the head is never oriented toward the rear. This consistent orientation made it possible to install the camera assembly at a single position on the ceiling, aligned with the expected location of the head.

**Figure 8:**
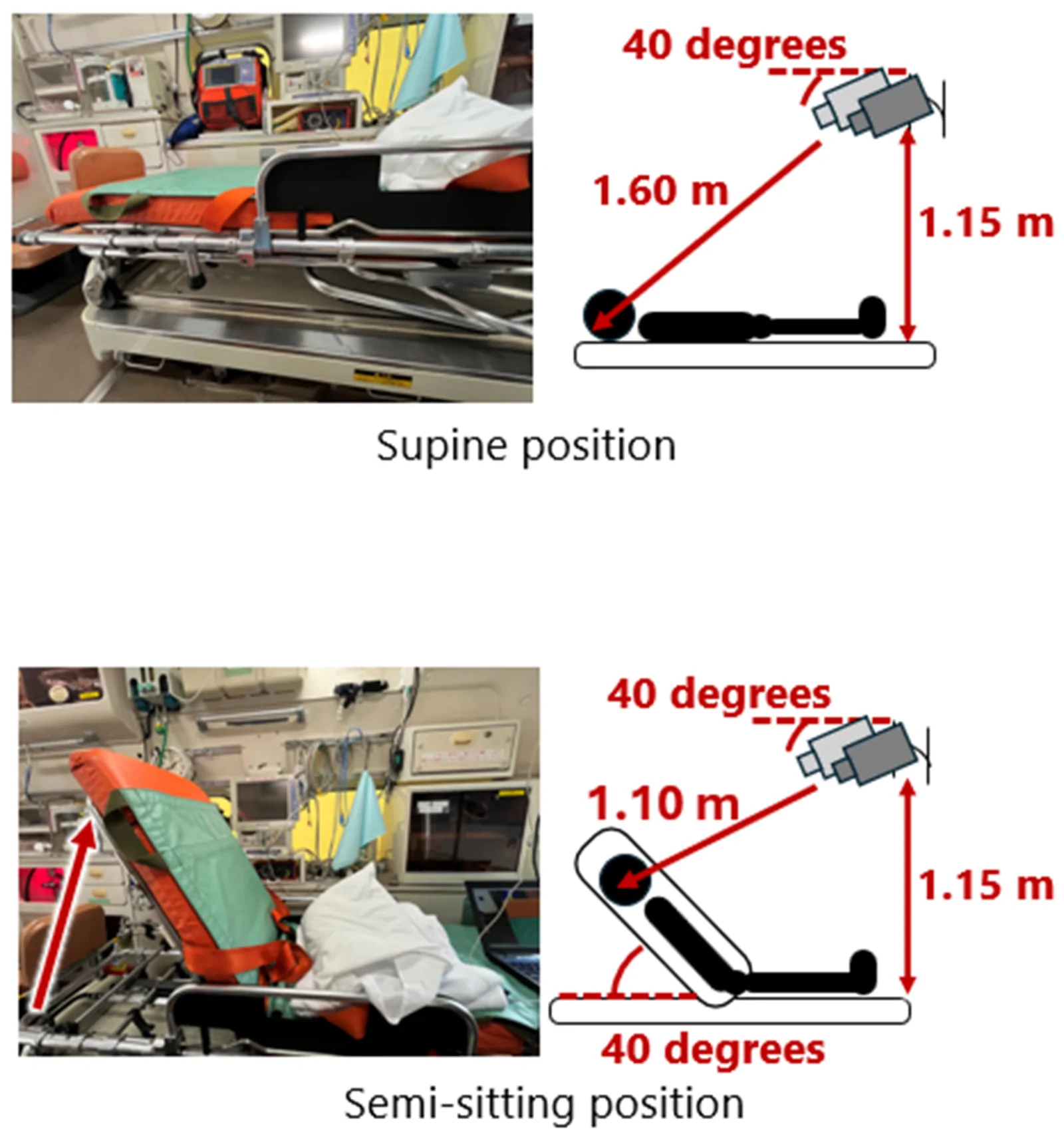
Experimental environment. Measurements were performed with the subject in both the supine and semi-sitting positions. The bed angle is adjustable; in this experiment, the semi-sitting position corresponded to a 40 degrees bed tilt.

The patient bed used in the ambulance is height and angle adjustable, with its inclination typically set according to patient preference during transport. To evaluate measurement performance under different postural conditions, data were collected with the bed in two configurations: (1) supine position, with a camera-to-bed distance of 1.60 m, and (2) semi-sitting position at a 40° incline, with a camera-to-bed distance of 1.10 m (Figure 8).

### 2.3. Measurement Protocol

For each subject, three 15-s measurements were recorded under each of three respiratory conditions: bradypnea, normal, and tachypnea corresponding to RR of 8, 15, and 30 breaths per minute (breaths/min), respectively, representing a wide range of RR that may be encountered during ambulance transport. A metronome sound was used to guide subjects in following the target RR during each trial. This resulted in a total of nine measurements (3 conditions × 3 trials) per participant for each posture.

A chest belt (GoDirect Respiratory Belt, Vernier Software & Technology, Beaverton, OR, USA) was attached to each subject to record chest wall movement, which served as the ground-truth reference for evaluating RR accuracy, independent of the instructed target rate.

During the preparation of this study (April 2025–August 2026), the authors used Claude (Anthropic), primarily Claude Sonnet 4.5, for the purpose of assisting in the development of code used for data analysis, as well as for language editing, including grammar, spelling, punctuation, and formatting. The authors have reviewed and edited the output and take full responsibility for the content of this publication.

## 3. Results

This section presents the evaluation results in three stages. We first assess the accuracy of the proposed patient posture-adaptive automatic image registration method. We then evaluate the accuracy of RR estimation using data collected inside a moving ambulance. Finally, we compare the proposed nostril detection method with conventional methods.

### 3.1. Evaluation of Image Registration

#### 3.1.1. Evaluation Methods of Patient Posture-Adaptive Automatic Image Registration Error

First, two corresponding image sets were prepared at distances of 1.10 m and 1.60 m, respectively, following the procedure described in Section 2.1.2.

**Figure 9:**
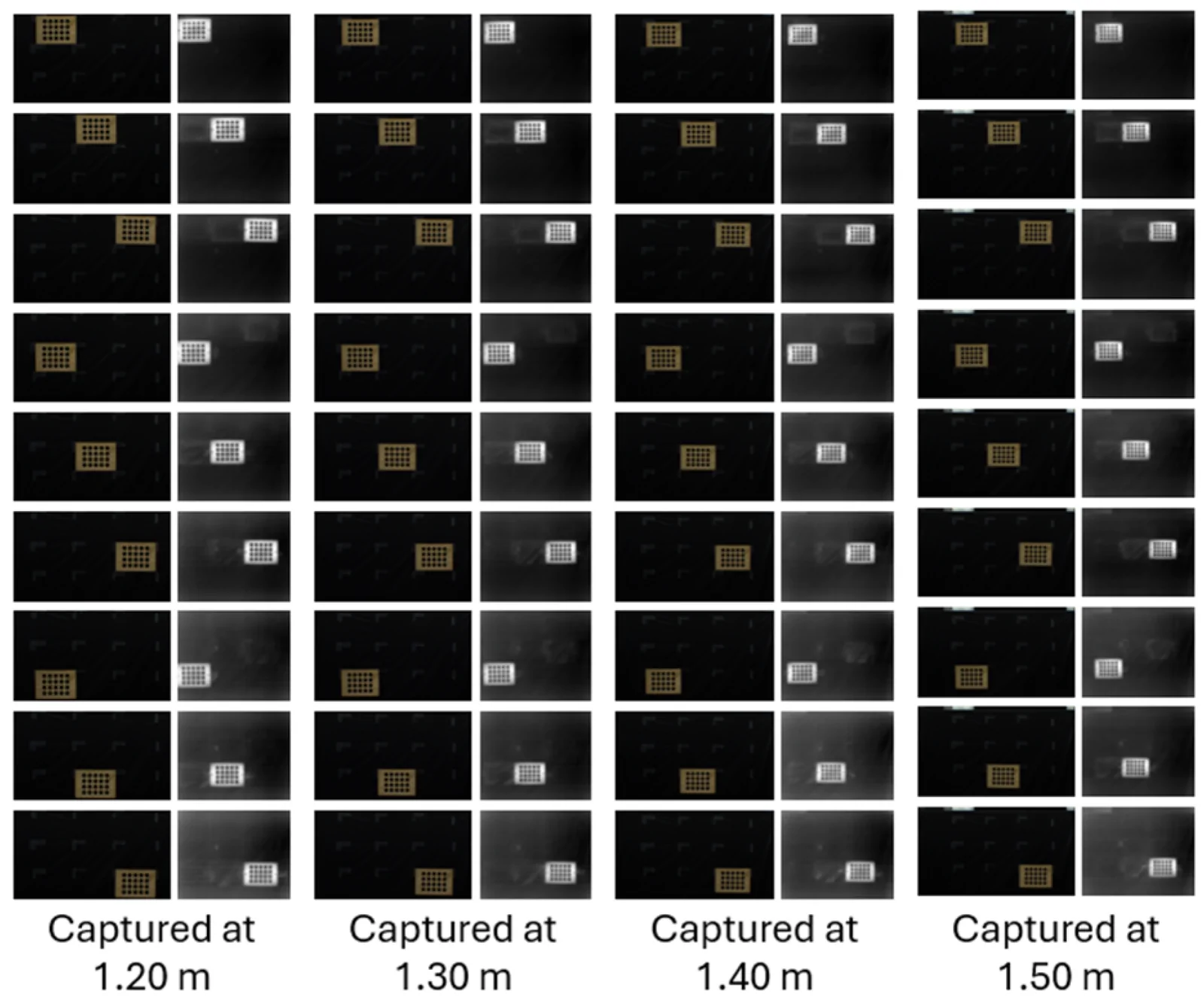
Calibration board images for evaluation of image registration. For each of the four distances (1.20 m, 1.30 m, 1.40 m, and 1.50 m), nine board images were captured with the board positioned perpendicular to the optical axis of the cameras. Each RGB–thermal image pair constitutes one set, resulting in a total of 36 sets. Within each set, the RGB and thermal images were registered according to the corresponding distance information.

For evaluation purposes, additional calibration board images were captured at 1.20 m, 1.30 m, 1.40 m, and 1.50 m (Figure 9). At each distance, nine board images were captured with the board positioned perpendicular to the optical axis of the cameras.

The proposed patient posture-adaptive automatic image registration method was then applied to each of the captured evaluation images at 1.20 m, 1.30 m, 1.40 m, 1.50 m, using the two prepared image sets at 1.10 m and 1.60 m. For each set, the image registration error was calculated as the average Euclidean distance between the corresponding grid point positions detected in the registered RGB image and those detected in the thermal image, using the six grid points out of 20 grid points located near the center of the board, as formulated in Equation (3).

(3) $$E=\frac{1}{N}\sum_{i=1}^{N}\sqrt{{\left(u_{i}^{R}-u_{i}^{T}\right)}^{2}+{\left(v_{i}^{R}-v_{i}^{T}\right)}^{2}},$$

where *N* denotes the number of grid points used for the error calculation (*N* = 6), $\left(u_{i}^{R},v_{i}^{R}\right)$ denotes the position of the *i*-th grid point detected in the registered RGB image, $\left(u_{i}^{T},v_{i}^{T}\right)$ denotes the position of the corresponding *i*-th grid point detected in the thermal image, and *E* denotes the resulting image registration error for that set.

This procedure was applied to all nine images at each of the four distances, yielding a total of 36 registration error measurements (9 positions × 4 distances) for evaluation.

#### 3.1.2. Results of Patient Posture-Adaptive Automatic Image Registration Error

Table 1 summarizes the mean, standard deviation (SD), maximum, and minimum image registration errors at each distance. The image registration error remained within 1 pixel at all other distances.

### 3.2. Evaluation of RR Estimation Inside an Ambulance Environment

#### 3.2.1. Evaluation Methods of RR Estimation

The proposed system was evaluated using RR data acquired in accordance with the experimental setup and measurement protocol described in Section 2.2 and Section 2.3. For each participant, three measurements were obtained under each combination of posture and respiratory condition. For each measurement, the absolute error between the estimated RR obtained from the proposed system and the ground-truth RR obtained from the chest band was calculated, and the mean of the three resulting errors was used as the mean absolute error (MAE), as defined in Equation (4), for evaluation.

(4) $$\mathrm{MAE}=\frac{1}{n}\sum_{i=1}^{n}\left|{\mathrm{RR}}_{e,i}-{\mathrm{RR}}_{g,i}\right|,$$

where *n* denotes the number of measurements (*n* = 3, corresponding to the three repeated trials for each combination of posture and respiratory condition); *i* denotes the index of each measurement; *RR_e_*_,*i*_ denotes the RR estimated by the proposed system for the *i*-th measurement; and *RR_g_*_,*i*_ denotes the corresponding ground-truth RR obtained from the chest band for the *i*-th measurement.

In addition to the MAE for each participant, the overall MAE for each combination of posture and respiratory condition was calculated across all participants, together with its 95% confidence interval, to quantify the uncertainty of the estimate. Measurement reliability and repeatability were further assessed based on the absolute error, rather than the predicted RR itself. This is because the ground-truth RR obtained from the chest belt varied slightly between the three trials, even under nominally identical conditions, reflecting natural variation in the subject’s respiration. If the predicted RR itself were used to assess repeatability, this natural variation in the ground-truth RR would be indistinguishable from variation caused by the system. By instead using the absolute error, which already accounts for the corresponding ground-truth value at each trial, the resulting variability reflects only the consistency of the system’s accuracy. At the individual level, the within-subject SD of the absolute error was calculated across the three trials for each subject and each combination of posture and respiratory condition. At the group level, this within-subject SD was pooled across all subjects for each combination of posture and respiratory condition, and the repeatability coefficient (RC) was calculated.

(5) $$\mathrm{RC}=1.96\sqrt{2}\mathrm{SD},$$

where *SD* denotes the pooled within-subject SD. The *RC* follows the conventional definition based on paired repeated measurements and indicates the value within which 95% of the difference between any two repeated measurements of absolute error is expected to fall.

#### 3.2.2. Results of RR Estimation

Table 2 shows the results for the supine position, and Table 3 shows the results for the semi-sitting position. Both tables present the MAE and the within-subject SD of the absolute error (in parentheses) for each participant under each respiratory condition. Overall, the results indicate that the accuracy of RR estimation was higher in the supine position than in the semi-sitting position, across all respiratory conditions. In addition, when comparing respiratory conditions, the estimation accuracy was lower for tachypnea than for bradypnea and normal. Several cases exhibited an MAE exceeding 3 breaths/min, which had an impact on the average MAE.

The overall MAE and its 95% confidence interval for each condition are summarized in Table 4. In the supine position, the 95% confidence intervals were [0.56, 1.33], [0.45, 0.73], and [1.05, 4.54] breaths/min for bradypnea, normal, and tachypnea, respectively; in the semi-sitting position, the corresponding intervals were [0.55, 1.62], [0.82, 2.99], and [1.57, 7.18] breaths/min. The wide confidence interval observed for tachypnea, particularly in the semi-sitting position.

Measurement repeatability, expressed as the pooled within-subject SD of the absolute error across the three trials, together with the RC, is also summarized in Table 4. Both indices followed the same trend as MAE: repeatability was lower during tachypnea (SD = 3.92 breaths/min, RC = 10.88 breaths/min, in the supine position; SD = 5.24 breaths/min, RC = 14.52 breaths/min, in the semi-sitting position) than during bradypnea or normal and was consistently lower in the semi-sitting position than in the supine position.

As shown in Table 2 and Table 3, the pooled within-subject SD and RC for tachypnea were disproportionately influenced by two individual cases: Subject C in the supine position (SD = 10.11 breaths/min) and Subject G in the semi-sitting position (SD = 12.80 breaths/min), which are examined individually in Section 4.4.

### 3.3. Comparison with Conventional Methods for Nostril Detection

#### 3.3.1. Evaluation Methods of Nostril Detection

The conventional and proposed nostril detection methods described in Section 2.1.3 were compared in terms of RR estimation accuracy. The absolute error between the estimated and ground-truth RR for each respiratory condition, and the Wilcoxon signed-rank test was used to assess whether there was a statistically significant difference between the conventional and proposed methods. In addition, Bland–Altman analysis [23] was performed separately for the conventional and proposed methods, comparing the estimated RR with the ground-truth RR, to evaluate the agreement between the estimated and reference values independently of statistical significance testing. The mean bias and 95% limits of agreement were calculated for each method.

#### 3.3.2. Results of Nostril Detection Method Comparison

**Figure 10:**
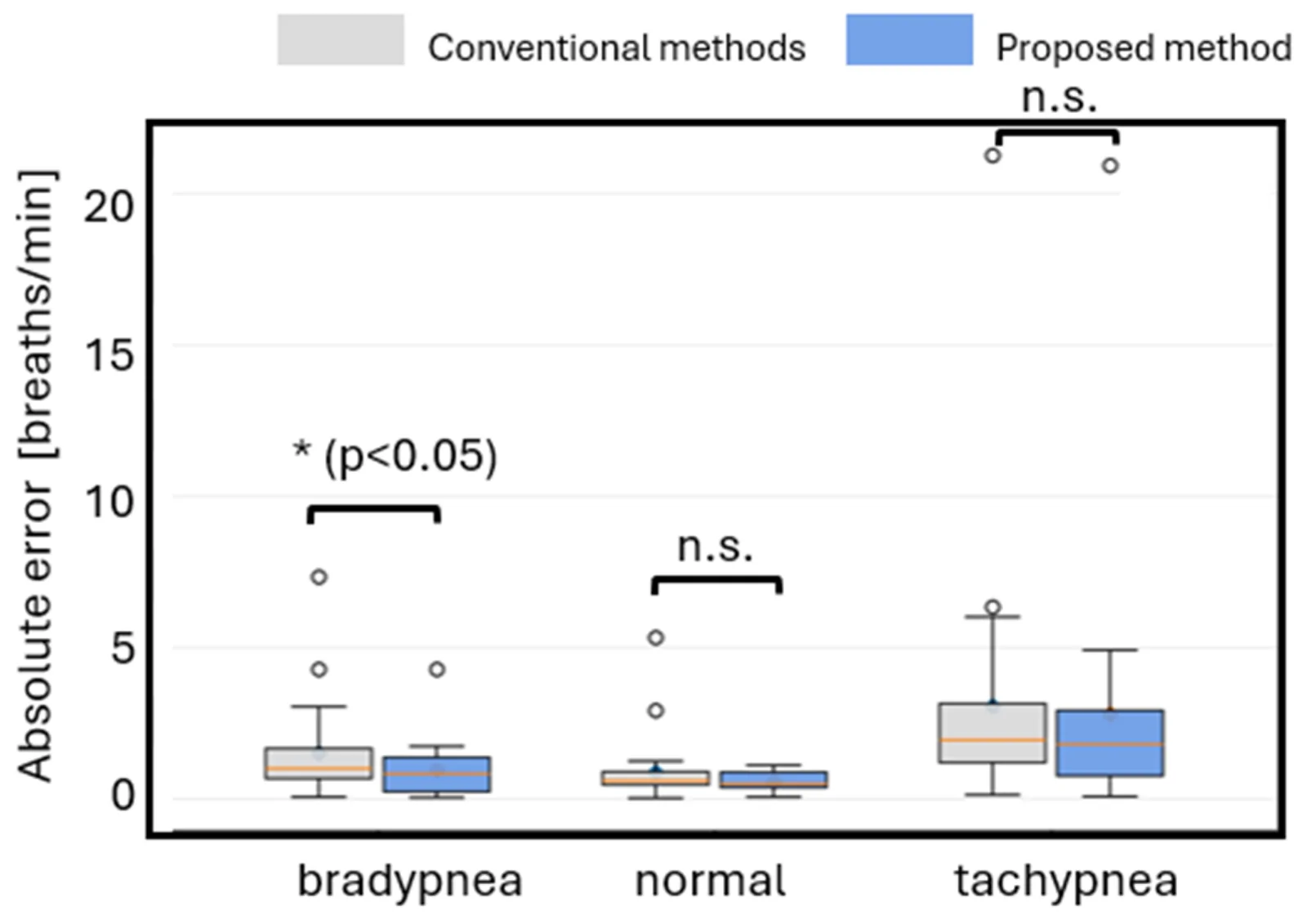
Comparison of conventional and proposed nostril detection methods in the supine position. The conventional method and the proposed method were compared for each respiratory condition (bradypnea, normal, and tachypnea), and statistical significance testing was performed. The line within each box indicates the median absolute error, and the diamond marker indicates the mean absolute error. The median absolute error for the conventional method was 1.00, 0.60, and 1.94 breaths/min for bradypnea, normal, and tachypnea, respectively, whereas the corresponding values for the proposed method were 0.82, 0.50, and 1.81 breaths/min.

**Figure 11:**
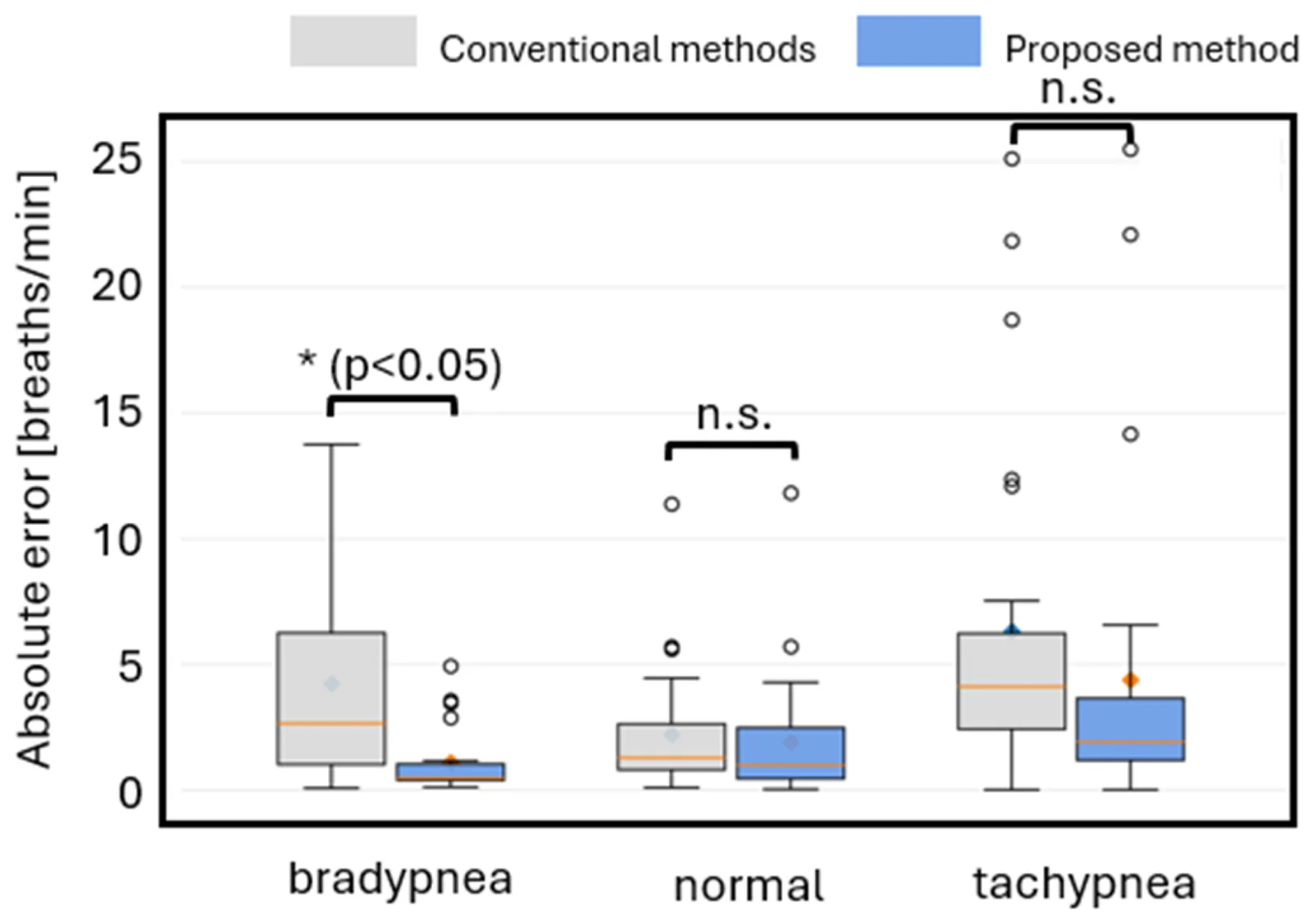
Comparison of conventional and proposed nostril detection methods in the semi-sitting position. The conventional method and the proposed method were compared for each respiratory condition (bradypnea, normal, and tachypnea), and statistical significance testing was performed. The line within each box indicates the median absolute error, and the diamond marker indicates the mean absolute error. The median absolute error for the conventional method was 2.65, 1.27, and 4.10 breaths/min for bradypnea, normal, and tachypnea, respectively, whereas the corresponding values for the proposed method were 0.46, 0.98, and 1.90 breaths/min.

The absolute error between the estimated and ground-truth RR for each respiratory condition is shown as a box plot; Figure 10 presents the results for the supine position, and Figure 11 presents the results for the semi-sitting position.

The results confirmed that estimation accuracy improved for all respiratory conditions in both the supine and semi-sitting positions. However, the Wilcoxon signed-rank test revealed a significant difference only for bradypnea, in both the supine and semi-sitting positions.

**Figure 12:**
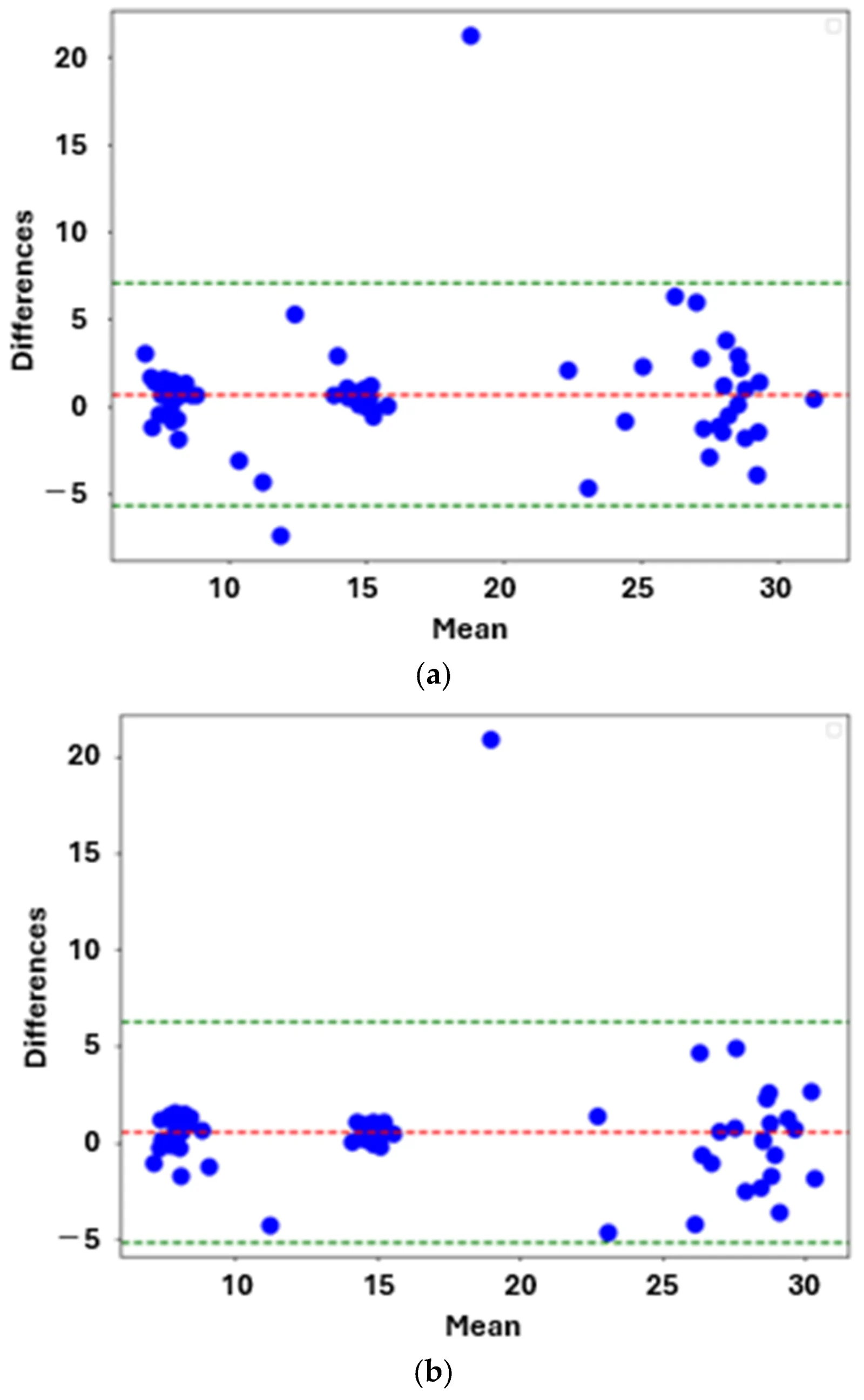
Bland–Altman plots comparing the estimated and ground-truth RR in the supine position: (**a**) conventional method (mean bias = 0.69 breaths/min; 95% limits of agreement = −5.68 to 7.06 breaths/min); (**b**) proposed method (mean bias = 0.54 breaths/min; 95% limits of agreement = −5.17 to 6.25 breaths/min). The horizontal axis shows the mean of the estimated and ground-truth RR, and the vertical axis shows subtraction ground-truth RR from estimated RR. The red dashed line indicates the mean bias, and the green dashed lines indicate the 95% limits of agreement.

**Figure 13:**
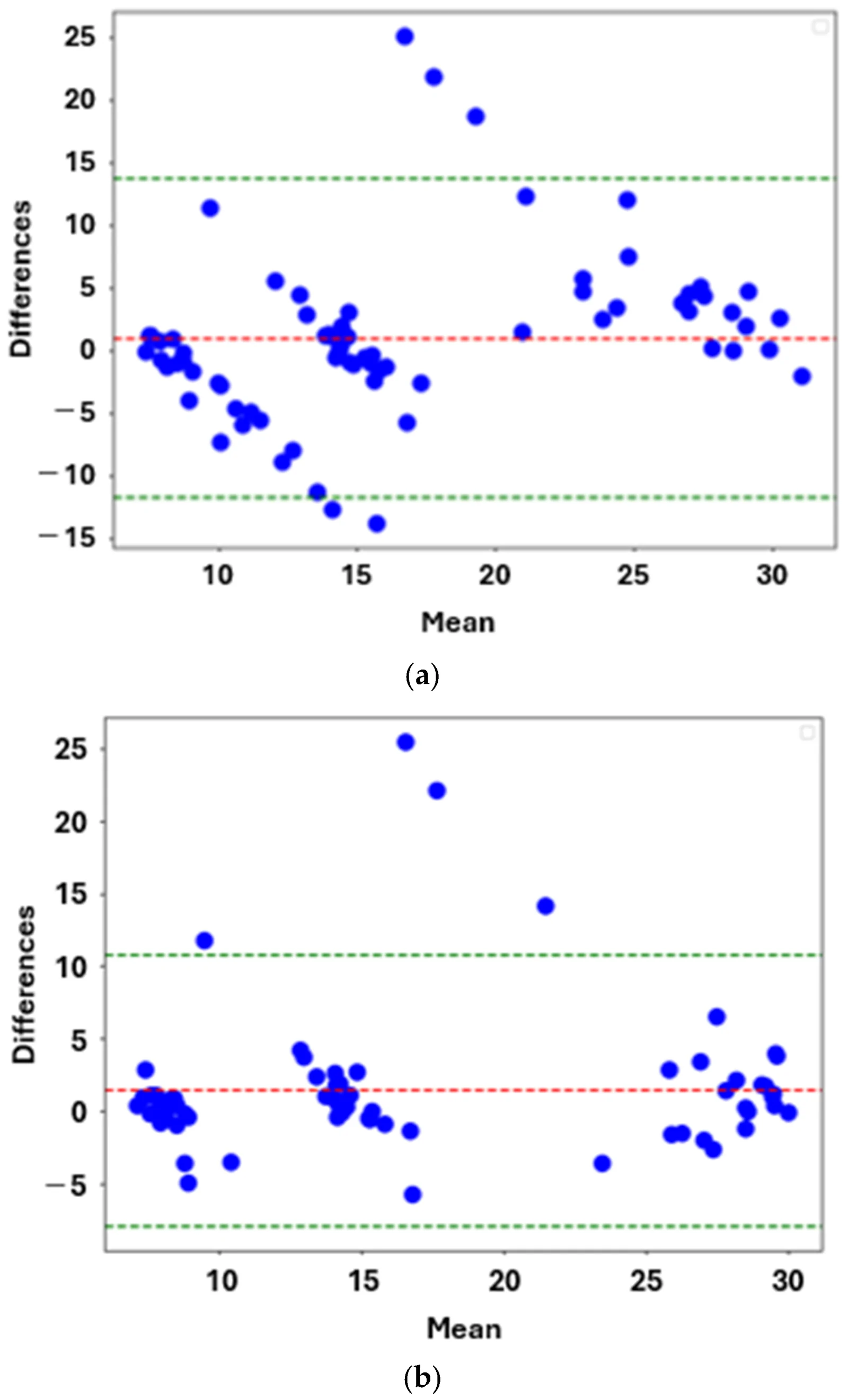
Bland–Altman plots comparing the estimated and ground-truth RR in the semi-sitting position: (**a**) conventional detection method (mean bias = 0.97 breaths/min; 95% limits of agreement = −11.77 to 13.71 breaths/min); (**b**) proposed method (mean bias = 1.43 breaths/min; 95% limits of agreement = −7.92 to 10.77 breaths/min). The horizontal axis shows the mean of the estimated and ground-truth RR, and the vertical axis shows subtraction ground-truth RR from estimated RR. The red dashed line indicates the mean bias, and the green dashed lines indicate the 95% limits of agreement.

Bland–Altman analysis was additionally performed to evaluate the agreement between the estimated and ground-truth RR for the conventional and proposed methods; Figure 12 presents the results for the supine position, and Figure 13 presents the results for the semi-sitting position. In the supine position, the mean bias was 0.69 breaths/min (95% limits of agreement: −5.68 to 7.06 breaths/min) for the conventional method and 0.54 breaths/min (95% limits of agreement: −5.17 to 6.25 breaths/min) for the proposed method. The proposed method showed both a smaller bias and narrower limits of agreement. In the semi-sitting position, the mean bias was 0.97 breaths/min (95% limits of agreement: −11.77 to 13.71 breaths/min) for the conventional method and 1.43 breaths/min (95% limits of agreement: −7.92 to 10.77 breaths/min) for the proposed method. Although the bias was slightly larger for the proposed method, the limits of agreement narrowed considerably, indicating reduced variability in the estimation error.

## 4. Discussion

In this study, we proposed a non-contact RR measurement method using RGB and thermal cameras that accommodate changes in patient posture inside an ambulance, and we confirmed its effectiveness through an experiment conducted inside a moving ambulance. Whereas previous studies were limited to experiments with subjects in a fixed posture under static conditions, the present study successfully validated the method across multiple postures under conditions in which the subject was subject to vibration-induced motion.

### 4.1. Image Registration

We first emphasize that the preparation of the two reference image sets described above is a one-off procedure, performed once before the cameras are installed in the ambulance. This is precisely the distinction from the calibration-based registration methods discussed in the Introduction, which require recalibration manually whenever the geometric conditions change. Once the reference image sets have been acquired, all subsequent operation proceeds automatically.

In the present evaluation of patient posture-adaptive automatic image registration, the range of distances examined was limited. However, comparable results to those obtained here are expected when calibration board images are prepared and registered under conditions similar to those used in this study, even at different distances. Furthermore, increasing the number of prepared corresponding image sets beyond two—for example, by preparing one set at every 0.10 m—is expected to further improve image registration accuracy. This improvement, however, comes at the cost of additional acquisition time, representing a trade-off between the desired accuracy and the time required for preparation. In the present study, despite using only two corresponding image sets, the image registration error remained within 1 pixel at any distance. Given that the size of a single nostril region is approximately 7 × 7 pixels, depending on the subject, posture, and distance from the camera, this level of registration error is considered sufficiently small for the intended application.

On the other hand, the image registration error improved with increasing distance. This can be attributed to the position of the calibration board within the camera’s field of view in the dataset used for evaluation (Figure 9). Because the board was fixed in place while the camera was moved farther away at each subsequent distance during dataset preparation, the board tended to be positioned closer to the periphery of the field of view at shorter distances. Peripheral regions of the image are more susceptible to residual distortion after camera calibration than central regions. Because image registration and grid point detection are performed on images retaining this residual distortion, a larger image registration error is likely to have occurred at shorter distances, where the board was positioned closer to the edge of the field of view.

It should also be noted that this image registration error was calculated from the discrepancy between the grid point positions detected in the RGB image and those detected in the thermal image. Because this calculation therefore also incorporates the grid point detection error, the actual image registration error may in fact be smaller than the value reported here.

Furthermore, accurate acquisition of distance information is also a key factor for the success of this registration method, since an inaccurate distance estimate would lead to the selection of an inappropriate homography matrix and consequently degrade image registration accuracy.

### 4.2. Effect of Posture on RR Estimation

Regarding the RR estimation results obtained with the proposed system, the higher accuracy observed in the supine position compared to the semi-sitting position is considered to be attributable to the effects of vibration inside the moving ambulance. In the semi-sitting position, the subject’s center of gravity is higher than in the supine position; through a lever-arm effect, head motion is amplified, and this is further compounded by mechanical play in the movable sections of the bed and a reduced sense of bodily stability resulting from decreased contact area with the bed surface. Consequently, the head is considered more likely to be subjected directly to vibration from vehicle motion and centrifugal force during turns, resulting in more pronounced head movement.

Because the RGB and thermal cameras operate independently, and although frame acquisition is instructed to occur simultaneously via software, a slight timing discrepancy between the two cameras can occur. Even when image registration itself is performed correctly, this timing discrepancy causes a mismatch in the frame acquisition timing, which in turn causes the shared nostril position information to be misaligned, resulting in the detection of a region unrelated to respiration. This effect is considered to have been more pronounced in the semi-sitting position, where vibration was greater.

### 4.3. Effect of Respiratory Condition on RR Estimation

Regarding the differences in estimation accuracy across respiratory conditions, the lower accuracy observed during tachypnea can be attributed to the RR calculation algorithm applied to the obtained respiratory waveform, as expressed in Equation (2). In this algorithm, as the denominator decreases, the sensitivity of the output to variations in the denominator increases, such that even a small change can produce a comparatively large error in the result. Because the denominator is smaller for tachypnea than for bradypnea or normal, the resulting error tends to be larger for tachypnea as an inherent consequence of the calculation.

### 4.4. Effect of Subject on RR Estimation

Regarding the differences by individual subject, in the supine position, Subject C exhibited a notably high MAE of 9.28 breaths/min during tachypnea. This was primarily attributable to a single measurement, among the three trials for Subject C under tachypnea, with an absolute error of 20.92 breaths/min, which substantially affected the overall MAE for this subject. This is considered to have resulted from non-uniformity correction occurring in the thermal camera during measurement, which caused the image to freeze for several frames and destabilized the measurement.

In the semi-sitting position, as noted above, the greater influence of vibration is one of the factors contributing to the reduced estimation accuracy. For example, Subject G, who showed a similarly elevated error in the semi-sitting position during tachypnea, was examined. The absolute errors for the three trials were 1.82, 25.5, and 22.1 breaths/min, respectively. The frame-to-frame displacement of the detected nostril position was examined for each trial (Table 5). The first trial, with a low error of 1.82 breaths/min, showed a small mean displacement (1.72 ± 2.86 pixel). In contrast, the second and third trials, with substantially higher errors, showed markedly larger mean displacements (7.67 ± 7.86 pixel and 6.35 ± 13.49 pixel, respectively), including a maximum single-frame displacement of 155.74 pixel in the third trial. This confirms that the elevated errors for Subject G coincided with substantially greater frame-to-frame movement of the nostril position, supporting vibration-induced head motion as a source of the reduced estimation accuracy. This within-subject variation across trials illustrates that the degree of vibration experienced was not constant even for the same subject, but varied depending on the timing of each measurement, for instance in relation to road conditions during transport. The same is likely to hold between subjects: because the timing of measurements differed among subjects, some subjects may have been more strongly affected by vibration than others, which would account for the inter-subject variability observed in the results.

### 4.5. Measurement Reliability

Measurement reliability was evaluated based on the absolute error rather than the predicted RR itself. This is because the ground-truth RR obtained from the chest belt varied slightly between the three trials, even under nominally identical conditions, reflecting natural variation in the subject’s respiration. If the predicted RR itself were used to assess repeatability, this natural variation in the ground-truth RR would be indistinguishable from variation caused by the system. By instead using the absolute error, which already accounts for the corresponding ground-truth value at each trial, the resulting variability reflects only the consistency of the system’s accuracy.

The pooled within-subject SD and the RC followed the same pattern as MAE, increasing during tachypnea and in the semi-sitting position; in the semi-sitting position during tachypnea, the RC reached 14.52 breaths/min, indicating that the accuracy of two repeated measurements under this condition could, in the worst case, differ by more than 14 breaths/min. However, these pooled values were disproportionately influenced by two individual cases with markedly larger within-subject SD during tachypnea: Subject C in the supine position (SD = 10.11 breaths/min) and Subject G in the semi-sitting position (SD = 12.80 breaths/min), consistent with the subject-specific factors discussed in Section 4.4 (thermal camera non-uniformity correction and vibration-induced frame misalignment, respectively). Excluding these two cases, the pooled within-subject SD for tachypnea decreased to 1.73 breaths/min (RC = 4.79 breaths/min) in the supine position and 2.07 breaths/min (RC = 7.83 breaths/min) in the semi-sitting position, indicating that the reduced repeatability observed during tachypnea in the pooled results reflects a small number of individual cases rather than a systematic limitation of the proposed method.

### 4.6. Effect of Proposed Nostril Detection Method

Finally, regarding the comparison of the nostril detection method before and after the proposed improvement, a statistically significant difference was observed only for bradypnea. However, the Bland–Altman analysis provided more direct evidence of improvement: the 95% limits of agreement narrowed with the proposed method in both postures, from 12.7 to 11.4 breaths/min in the supine position and, more markedly, from 25.5 to 18.7 breaths/min in the semi-sitting position, indicating reduced variability in the estimation error. RR estimation accuracy also improved across all respiratory conditions, indicating that the proposed improvement was of practical value.

It should be noted that the benefit of pinpoint nostril detection is expected to manifest fully only when image registration is performed accurately. As discussed in Section 4.2, the timing discrepancy between the two cameras can cause misalignment of the shared nostril position information, which may have obscured the statistical significance of the improvement in some respiratory conditions.

### 4.7. Limitations

This study has several limitations. First, the number of subjects remains small (*n* = 8), making it difficult to fully identify the subject-specific factors underlying the differences in estimation accuracy. In addition, the cohort was demographically homogeneous, consisting of healthy adults aged 21–23 years. As this is, to our knowledge, the first evaluation of non-contact RR measurement inside a moving ambulance, priority was given to establishing the core technical feasibility of the proposed method using a small, controlled cohort before extending to a larger and more diverse population. In particular, the target population during ambulance transport includes elderly patients, whose facial characteristics, nasal airflow, and breathing patterns may differ substantially from those of the present cohort. Validation across a larger and demographically broader cohort is therefore an essential direction for future work.

Second, each condition was measured three times per subject, using a 15 s window. This window is considered appropriate, as it reflects the interval over which RR is conventionally measured in emergency settings, and the number of repetitions was adopted to keep the total protocol duration inside the ambulance within an acceptable duration for each subject. Although a repeatability analysis based on these three trials is presented in Section 4.5, this number of repetitions remains limited for a statistically robust assessment of reliability. A larger number of repetitions should therefore be incorporated into future studies to enable a more rigorous evaluation.

Third, as noted above, because frame acquisition of the two cameras is controlled via software instruction, frame synchronization has a substantial impact on accuracy in environments with pronounced vibration. Accordingly, future work should focus on increasing the number of subjects and achieving strict frame synchronization through the use of an external hardware trigger.

Fourth, a universally accepted definition of a clinically acceptable RR estimation error has not been established in the literature. A review of the non-contact and wearable RR monitoring literature found that many studies do not clearly specify such a threshold. Where a threshold is specified, for example, Maurya et al. [14], one of the RGB–thermal registration studies, define the tolerance level as ±15% of the breathing rate, based on medical practice. Following this literature review, we consulted with emergency physicians at our collaborating institution, who confirmed that no single, widely accepted threshold for clinically significant RR estimation error has been established. Under the relative criterion of Maurya et al., the MAE observed in the present study, including during tachypnea (2.80 breaths/min in the supine position and 4.38 breaths/min in the semi-sitting position, against a tolerance of ±4.5 breaths/min), remained within tolerance across all conditions. We nonetheless consider it important to collect further data across a denser range of RR, particularly at higher rates, to more thoroughly evaluate the robustness of the system. However, sustaining higher RR for the required duration imposes a greater physical burden on subjects than the conditions tested in the present study. Collecting data across a denser range of RR remains an important direction for future work.

Fifth, because nostril detection relies on the RGB image, the performance of the proposed system is dependent on lighting conditions. Although the present experiments were conducted under naturally varying daytime illumination, cabin lighting inside an ambulance may occasionally be turned off during transport at the patient’s request, and performance under such dark conditions was not evaluated in this study. Because the respiratory signal itself is derived from long-wave infrared emission and is therefore independent of visible illumination, this limitation is expected to affect only the RGB-based nostril detection step rather than the underlying measurement principle. Evaluating and, if necessary, adapting the system for dark conditions is an important direction for future work.

Finally, because the proposed system estimates RR based on temperature changes at the nostrils, it cannot obtain a respiratory waveform in situations where the nostrils are not visible, as is the case for patients wearing an oxygen mask. However, this limitation is expected to have limited practical consequence, because patients who already require oxygen therapy are typically those presenting with visibly severe conditions, and such patients generally already receive closer clinical attention and continuous monitoring from the accompanying emergency personnel. The primary value of the proposed system lies instead in monitoring patients with mild-to-moderate conditions, who do not initially present with signs warranting oxygen therapy but may deteriorate suddenly and unpredictably during transport, and for whom such close continuous monitoring is not otherwise routinely provided.

## 5. Conclusions

In this article, we proposed a non-contact RR measurement system using RGB and thermal cameras for use inside an ambulance. Specifically, we proposed a method for patient posture-adaptive automatic image registration between the two cameras that accommodates changes in patient posture, together with a nostril detection method. The effectiveness of the proposed system was confirmed through measurements involving eight subjects, conducted under two postures (supine and semi-sitting) and three respiratory conditions (bradypnea, normal, and tachypnea). These results, obtained under conditions simulating a moving ambulance with various respiratory conditions and postural changes, are expected to serve as an important benchmark for future RR measurement inside ambulances and represent a meaningful step toward clinical application. Future work will focus on two main directions: expanding the evaluation to a larger and more diverse cohort under a wider range of conditions, including additional RR and lighting environments, and improving the technical robustness of the system, particularly through stricter frame synchronization using an external hardware trigger.

## Figures and Tables

**Table 1:** Results of automatic image registration error at 1.20 m, 1.30 m, 1.40 m, 1.50 m. Mean, standard deviation (SD), maximum (Max), and minimum (Min) values are shown.

Distance	Mean Error [Pixel]	SD [Pixel]	Max Error [Pixel]	Min Error [Pixel]
1.20 m	0.77	0.23	0.99	0.36
1.30 m	0.54	0.25	0.90	0.15
1.40 m	0.44	0.13	0.71	0.27
1.50 m	0.27	0.08	0.41	0.18

**Table 2:** Results of MAE and within-subject SD in supine position. SD denotes the within-subject SD of the absolute error, shown in parentheses.

Subject	Absolute Error [Breaths/min]		
	Bradypnea Mean (SD)	Normal Mean (SD)	Tachypnea Mean (SD)
Subject A	1.93 (2.10)	0.36 (0.47)	1.71 (0.88)
Subject B	0.68 (0.48)	0.76 (0.30)	1.14 (1.07)
Subject C	0.71 (0.62)	0.60 (0.22)	9.28 (10.11)
Subject D	1.06 (0.80)	0.80 (0.35)	2.35 (2.28)
Subject E	0.80 (0.68)	0.57 (0.44)	2.14 (2.18)
Subject F	1.14 (0.56)	0.50 (0.19)	1.90 (0.63)
Subject G	0.67 (0.67)	0.34 (0.09)	1.84 (1.56)
Subject H	0.57 (0.79)	0.76 (0.46)	2.03 (2.49)
Average	0.95 (0.84)	0.59 (0.32)	2.80 (2.65)

**Table 3:** Results of MAE and within-subject SD in semi-sitting position. SD denotes the within-subject SD of the absolute error, shown in parentheses.

Subject	Absolute Error [Breaths/min]		
	Bradypnea Mean (SD)	Normal Mean (SD)	Tachypnea Mean (SD)
Subject A	1.53 (1.68)	1.74 (1.76)	1.93 (1.30)
Subject B	0.56 (0.53)	0.72 (0.41)	0.52 (0.82)
Subject C	0.45 (0.02)	1.73 (2.20)	3.81 (2.75)
Subject D	0.84 (0.36)	1.52 (1.14)	6.77 (6.46)
Subject E	0.29 (0.16)	2.71 (2.84)	1.32 (0.82)
Subject F	3.10 (2.09)	1.14 (0.72)	1.95 (0.58)
Subject G	1.11 (1.52)	4.99 (6.01)	16.47 (12.80)
Subject H	0.78 (0.28)	0.67 (1.01)	2.24 (1.76)
Average	1.08 (0.83)	1.90 (2.01)	4.38 (3.41)

**Table 4:** Summary of MAE with 95% confidence intervals, together with measurement repeatability, for each respiratory condition in the supine and semi-sitting positions. Repeatability is expressed as the pooled within-subject SD of the absolute error across all subjects, and the corresponding RC.

Posture	Condition	MAE [Breaths/min]	95% CI	Pooled Within- Subject SD [Breaths/min]	RC [Breaths/min]
Supine	Bradypnea	0.95	[0.56, 1.33]	0.96	2.68
Supine	Normal	0.59	[0.45, 0.73]	0.34	0.95
Supine	Tachypnea	2.80	[1.05, 4.54]	3.92	10.88
Semi-sitting	Bradypnea	1.08	[0.55, 1.62]	1.12	3.10
Semi-sitting	Normal	1.90	[0.82, 2.99]	2.63	7.28
Semi-sitting	Tachypnea	4.38	[1.57, 7.18]	5.24	14.52

**Table 5:** Frame-to-frame displacement of the detected nostril position for Subject G (semi-sitting, tachypnea). Absolute error, mean, standard deviation (SD), maximum (Max) values are shown.

Trial	Absolute Error [Breaths/min]	Mean [Pixel]	SD [Pixel]	Median [Pixel]	Max [Pixel]
Trial 1	1.82	1.72	2.86	0.48	26.44
Trial 2	25.49	7.67	7.86	5.01	41.51
Trial 3	22.10	6.35	13.49	2.66	155.74

## Data Availability

The data presented in this study are not publicly available due to ethical restrictions, as the dataset contains video recordings of identifiable human participant. Researchers wishing to access the data may contact the corresponding author, and requests will be considered on a case-by-case basis.
